# Health needs and care seeking behaviours of Yazidis and other minority groups displaced by ISIS into the Kurdistan Region of Iraq

**DOI:** 10.1371/journal.pone.0181028

**Published:** 2017-08-16

**Authors:** Valeria Cetorelli, Gilbert Burnham, Nazar Shabila

**Affiliations:** 1 Center for Humanitarian Health, Johns Hopkins Bloomberg School of Public Health, Baltimore, Maryland, United States of America; 2 Middle East Centre, London School of Economics and Political Science, London, United Kingdom; 3 Department of Community Medicine, College of Medicine, Hawler Medical University, Erbil, Kurdistan Region, Iraq; Queensland University of Technology, AUSTRALIA

## Abstract

**Background:**

During the summer of 2014, ISIS overran Nineveh governorate in Northern Iraq. Yazidis and other religious minorities were subjected to brutal attacks and forced to seek refuge into the neighbouring Kurdistan Region, where they remain living in local communities or in camps. This survey provides a population-based assessment of the health needs and care seeking behaviours of Yazidis and other groups currently residing in camps.

**Methods:**

The survey covered 13 camps managed by the Kurdish Board of Relief and Humanitarian Affairs. A systematic random sample of 1,300 households with a total of 8,360 members were interviewed between November and December 2015. Participants were asked if any household members had needed care for a health condition in the two weeks preceding the survey, and whether care was obtained from the camp primary health care centre, an outside public hospital or a private clinic. If care was received, the out-of-pocket payment was recorded; otherwise, the reason for not seeking care was queried.

**Results:**

In 33.9% (CI: 31.0–37.0) of households one or more members had needed care for a health condition in the two weeks preceding the survey. The most likely to have needed care were older persons (18.5%; CI: 13.6–24.6) and infants (18.0%; CI: 11.6–26.8). The reported health conditions revealed a complex picture of communicable and non-communicable diseases as well as mental health problems and physical injuries. Care was primarily sought from private clinics (41.8%; CI: 36.4–47.4) or public hospitals (27.3%; CI: 22.6–32.7) rather than from the camp primary health care clinics (23.6%; CI: 19.5–28.2). The mean out-of-pocket payment for care received was nearly 3 times higher in public hospitals than in the camp primary health care clinics and nearly 11 times higher in private clinics. Cost was the main perceived barrier to obtaining health services.

**Conclusion:**

Demand for health services was high among Yazidis and other minorities living in camps. Private services were preferred in spite of the tenuous economic circumstances of displaced households. Declines in public sector funding may further restrict access from camp clinics stressing the need for alternative access strategies.

## Introduction

There are currently more people displaced by conflict and persecution than at any other time since World War II [1]. Ensuring that their health needs are adequately addressed is an increasing challenge for humanitarian agencies and host country governments. Until recently, camp-based health care strategies have focused on communicable diseases and maternal and child care [2]. While traditional health priorities remain relevant, demographic and lifestyle changes are shifting the disease burden towards non-communicable diseases that are more complicated and costly to manage. Violence and displacement also result in physical injuries and mental health problems that may require long-term specialised care [3].

The seizure of one-third of Iraq’s territory by the so-called Islamic State of Iraq and Syria (ISIS) during the course of 2014 and the subsequent military actions caused a massive displacement crisis in the country [4]. Displacement flows closely followed a sectarian pattern. Between June and August 2014, ISIS overran Nineveh governorate in northern Iraq that has historically been home to most of Iraq’s religious minorities, including Yazidis, Assyrian and Chaldean Christians, Sabaean-Mandaeans, Turkmen, Shabak and Kaka’i. These minorities were systematically targeted by ISIS in a violent campaign to “purify” the region of non-Islamic influences [5,6]. Most members of targeted minorities sought refuge in the neighbouring Duhok governorate within the Kurdistan Region of Iraq. While some managed to rent houses or stay with host families, others had no option but to find temporary shelter in schools, community spaces, or unfinished buildings under untenable conditions, before being gradually transferred to newly constructed camps.

By the end of 2015, a Board of Relief and Humanitarian Affairs (BRHA) in Duhok was managing 13 camps sheltering around 200,000 internally displaced persons (IDPs) from Nineveh governorate [7,8]. The vast majority of IDPs residing in BRHA camps are Yazidis, a religious minority whose beliefs and practices spans thousands of years. Originally from the area of Mount Sinjar in Nineveh governorate, the Yazidis have long been one of the most vulnerable and impoverished communities in Iraq. After having suffered from decades of discrimination, marginalisation and neglect under Saddam Hussein’s regime, in recent years they have experienced increasing persecution by Sunni extremists [9]. When ISIS assaulted Sinjar City and surrounding villages in August 2014, the Yazidis were singled out for executions and kidnappings and besieged for days on Mount Sinjar without access to water, food or medical care in temperatures rising above 50 degrees Celsius [10]. The particular circumstances of their displacement and their limited pre-existing ties with local communities in Kurdistan have rendered them especially in need of external assistance [6].

Ensuring that IDPs have access to adequate health services is a persisting concern in BRHA camps. Each camp is equipped with a Primary Health Care Clinic (PHCC) providing basic preventive and curative services under the management of the Duhok Directorate of Health and partner NGOs. However, most PHCCs are only open in the mornings, are staffed only by general medical officers and often lack adequate medicines for non-communicable diseases. As a result, many of the displaced bypass the PHCC to seek health care from public hospitals or private clinics outside camps and incur out-of-pocket expenses [7,8]. We conducted this study to characterise the health needs of in-camp IDPs and understand how they sought care. The goal was to provide the Kurdistan regional government and health care providers with information which can help deliver more effective care to this displaced population with limited resources.

## Methods

This study covered the 13 BRHA camps hosting Yazidis and other groups displaced by ISIS from Nineveh governorate: Bajed Kandala, Bardarash, Bersive, Chamisku, Dawdiya, Essian, Garmawa, Karbato, Khanke, Mamilian, Rwanga, Shariya, and Sheikhan. To ensure that all camps were sufficiently represented, we selected a stratified systematic random sample of 100 households in each camp, yielding a total of 1,300 households. For each camp, we determined a sampling interval *k* as the ratio of camp size to sample size. We chose a random number from 1 to *k* to identify a starting household and selected every *k*th household thereafter.

Besides collecting information about the current household composition and any killings and kidnappings of household members by ISIS [10], the questionnaire contained a module on health needs and care seeking behaviours. Participants were asked if any household members had needed care for a health condition in the two weeks preceding the interview. A two-week recall period is commonly used for health-related questions in household surveys, including in the USAID-supported Demographic and Health Surveys and the UNICEF-supported Multiple Indicator Cluster Surveys. The reported health conditions were classified into the following categories: respiratory infection, diarrhoea, fever, skin problem, urinary tract infection, gynaecological problem, eye problem, ear problem, chronic health condition, mental health problem, injury and other. Interviewees were then asked whether care was sought in the camp PHCC, a public hospital or a private clinic. If care was received, the out-of-pocket payment for consultation, laboratory tests and medicines was recorded. Otherwise, the reason for not seeking care for this condition was queried.

The questionnaire was first drafted in English, translated to Arabic and back translated to English. A focus group was organised at the Hawler Medical University to discuss key issues related to content, translation and interview flow. We revised the questionnaire based on the focus group discussion and agreed upon a consensus translation. This version of the questionnaire was pilot tested in 100 households in Shariya camp in October 2015 and final changes were made based on data evaluation and feedback from interviewers.

The field team consisted of four pairs of local Yazidi interviewers, with one male and one female in each pair, and one survey supervisor. The interviewers were given a three-day training session concerning the questionnaire, sampling methods, data collection using tablets, interview techniques and basic principles of human subject protection. Fieldwork took place between November and December 2015. Interviews were conducted with the household head or a responsible adult in the household. In order to improve the accuracy of response, efforts were made to involve household adults other than the primary respondent in the interview if questions pertained to them. The interviewers obtained verbal informed consent from all participants after reading a consent form explaining the purpose of the survey, its confidentiality and the voluntary nature of participation. In case the selected shelter was empty, responsible persons were not available to interview or refused to participate, the interviewers were instructed to conduct an interview with the household living in the nearest shelter. To protect the anonymity of respondents, no unique identifiers were recorded.

Data were collected on tablets using the Magpi platform (DataDyne LLC, Washington DC). Stata 14 (College Station, Texas) was used to perform the analysis. Descriptive statistics with accompanying 95% confidence intervals were calculated, adjusting for sampling design [11]. Differences in proportions and means were assessed using the chi-squared and t-test methods. The proportions of IDPs needing care in the two weeks preceding the survey and their reported health conditions were examined separately for infants under one year, young children aged 1–4 years, older children aged 5–14 years, male and female adults aged 15–59 years, and older persons aged 60 years and above. The proportions for those receiving care and the type of facility where care was sought were also assessed according to age groups and health conditions. The mean out-of-pocket expenses for care received by type of facility were converted to USD at an exchange rate of 1,167 IQD per USD [12].

The study received ethical approval from the Middle East Centre Committee of the London School of Economics and Political Science, London, UK and the Ethics Committee of the Hawler Medical University, Erbil, Kurdistan Region of Iraq. Permission to conduct the survey was granted by BRHA, Duhok, Kurdistan Region of Iraq. Analysis of the data was declared exempt by the Institutional Review Board of the Johns Hopkins Bloomberg School of Public Health, Baltimore, MD, USA.

## Results

Of the 1,300 selected households, 93 (7.2%) were replaced with households living in the nearest shelter because responsible adults were absent (6.5%) or they refused to participate in the survey (0.7%). The interviewed households included a total of 8,360 members (Table 1). The average household size was 6.9 (CI: 6.7–7.1). The proportion of males and females was 50.5% (CI: 49.5–51.5) and 49.5% (CI: 48.5–50.6) respectively; 42.9% (CI: 41.4–44.4) of household members were children under 15 years of age. All households were displaced from their homes in Nineveh governorate during the ISIS attacks of June-August 2014, and approximately 80.0% (CI: 77.9–81.9) were Yazidi. In the majority of households (54.5%; CI: 51.5–57.5) no one was formally employed at the time of the survey; casual labour, savings and humanitarian assistance constituted the main sources of support.

**Table 1:** Sample characteristics.

	Unweighted N
**Total household members**	8,360
Infants (<1)	159
Young children (1–4)	997
Older children (5–14)	2,524
Male adults (15–59)	2,189
Female adults (15–59)	2,201
Elderly (60+)	309
**Needed care in the last two weeks**	499
Respiratory infection	99
Diarrhoea	63
Fever	28
Skin problem	28
Urinary tract infection	62
Gynaecological problem	16
Eye problem	26
Ear problem	13
Chronic health condition	26
Mental health problem	49
Injury	16
Other	73
**Received care**	455
Camp PHCC	144
Public hospital	121
Private clinic	190

In 33.9% (CI: 31.0–37.0) of households one or more members had needed care for some health condition in the two weeks preceding the survey. Overall, the most prevalent health condition reported was respiratory infection (17.6%; CI: 14.1–21.7), followed by diarrhoea (13.5%; CI: 10.2–17.1), urinary tract infection (12.9%; CI: 9.7–17.1), mental health problem (9.0%; CI: 6.5–12.4), skin problem (6.3%; CI: 4.1–9.6), eye problem (6.2%; CI: 4.0–9.6), chronic health condition (6.2%; CI: 4.0–9.5), fever (5.0%; CI: 3.2–7.6), gynaecological problem (4.6%; CI: 2.7–7.8), injury (2.6%; CI: 1.5–4.5) and ear problem (1.8%; CI: 0.9–3.5).

The need for care varied significantly by age group (*p*<0.001), from 18.0% (CI: 11.6–26.8) among infants, to 7.8% (CI: 6.0–10.1) among young children, 3.4% (CI: 2.6–4.3) among older children, 4.4% (CI: 3.4–5.5) among male adults, 9.3% (CI: 7.9–10.9) among female adults, and 18.5% (CI: 13.6–24.6) among older persons (Table 2). The reasons for needing care also differed significantly by age groups (*p*<0.001). The three most commonly reported reasons among infants were respiratory infection (33.3%; CI: 15.2–58.1), diarrhoea (33.2%; CI: 15.2–58.0) and skin problem (15.2%; CI: 4.4–41.1). Among young children, common reasons were respiratory infection (35.1%; CI: 23.5–48.7), diarrhoea (27.8%; CI: 17.1–41.9) and fever (10.7%; CI: 5.1–21.2). Among older children, common reasons were respiratory infection (18.6%; CI: 10.7–30.3), eye problem (12.2%; CI: 4.7–28.1) and diarrhoea (10.7%; CI: 5.2–20.8). In male adults, frequent reasons for needing care were urinary tract infection (17.3%; CI: 9.3–29.9), diarrhoea (13.1%; CI: 6.6–24.4) and respiratory infection (12.3%; CI: 6.8–21.2). The most frequent conditions among female adults were urinary tract infection (19.0%; CI: 13.4–26.3), mental health problem (15.0%; CI: 9.7–20.4) and gynaecological problem (12.5%; CI: 7.4–20.4). In persons aged 60 and above the frequently reported reasons for needing care were eye problem (21.4%; CI: 10.4–39.0), chronic health condition (16.8%; CI: 7.7–32.8) and respiratory infection (15.8%; CI: 7.6–30.1).

**Table 2:** Health needs and care seeking behaviours in the two weeks preceding the survey.

	Infants (<1)	Young children (1–4)	Older children (5–14)	Male adults (15–59)	Female adults (15–59)	Elderly (60+)
	% (CI)	% (CI)	% (CI)	% (CI)	% (CI)	% (CI)
**Needed care in the last two weeks**	18.0 (11.6–26.8)	7.8 (6.0–10.1)	3.4 (2.6–4.3)	4.4 (3.4–5.5)	9.3 (7.9–10.9)	18.5 (13.6–24.6)
**Reasons for needing care**						
Respiratory infection	33.3 (15.2–58.1)	35.1 (23.5–48.7)	18.6 (10.7–30.3)	12.3 (6.8–21.2)	10.5 (6.5–16.5)	15.8 (7.6–30.1)
Diarrhoea	33.2 (15.2–58.0)	27.8 (17.1–41.9)	10.7 (5.2–20.8)	13.1 (6.6–24.4)	8.9 (4.9–15.4)	3.5 (0.9–12.4)
Fever	13.9 (5.0–33.1)	10.7 (5.1–21.2)	7.3 (3.1–16.2)	2.8 (0.8–9.6)	2.9 (1.2–7.0)	–
Skin problem	15.2 (4.4–41.1)	8.2 (2.6–22.9)	13.9 (6.7–26.6)	3.5 (1.0–11.8)	4.0 (1.8–8.5)	2.2 (0.3–14.4)
Urinary tract infection	–	3.2 (0.8–12.0)	10.6 (4.9–21.4)	17.3 (9.3–29.9)	19.0 (13.4–26.3)	9.5 (3.0–26.6)
Gynaecological problem	–	–	–	–	12.5 (7.4–20.4)	–
Eye problem	–	–	12.2 (4.7–28.1)	5.8 (2.5–6.4)	2.8 (1.2–6.4)	21.4 (10.4–39.0)
Ear problem	–	2.9 (0.8–10.1)	1.0 (0.2–4.5)	2.6 (0.1–7.5)	2.2 (0.7–6.6)	–
Chronic health condition	–	1.7 (0.2–11.3)	2.5 (0.1–10.0)	9.8 (3.8–23.2)	5.2 (2.7–9.8)	16.8 (7.7–32.8)
Mental health problem	–	2.6 (0.6–10.6)	8.0 (3.4–17.6)	7.6 (3.7–14.7)	15.0 (9.7–20.4)	6.2 (2.0–17.9)
Injury	–	–	1.6 (0.4–6.7)	9.8 (4.9–18.7)	1.8 (0.6–4.9)	1.1 (0.1–7.3)
Other	4.4 (0.6–25.6)	7.7 (3.4–16.5)	13.7 (6.7–26.0)	15.4 (9.1–25.1)	15.2 (10.1–22.2)	23.4 (12.6–39.4)
**Received care**						
No	–	8.4 (2.8–22.3)	8.4 (3.7–18.0)	9.0 (4.5–17.1)	7.8 (4.6–12.9)	4.1 (0.9–16.5)
Camp PHCC	32.1 (14.7–56.3)	28.3 (18.5–40.6)	41.4 (29.3–54.7)	24.5 (15.9–35.7)	16.5 (11.3–23.5)	13.8 (6.3–27.6)
Public hospital	23.5 (8.7–49.5)	44.9 (31.8–58.8)	23.5 (13.6–37.5)	17.0 (9.4–28.8)	23.7 (21.5–37.3)	21.8 (10.9–38.8)
Private clinic	44.5 (23.7–67.3)	18.4 (9.9–31.7)	26.7 (16.1–37.5)	49.6 (37.7–61.6)	46.9 (38.7–55.3)	60.3 (43.3–75.2)

**Fig 1:**
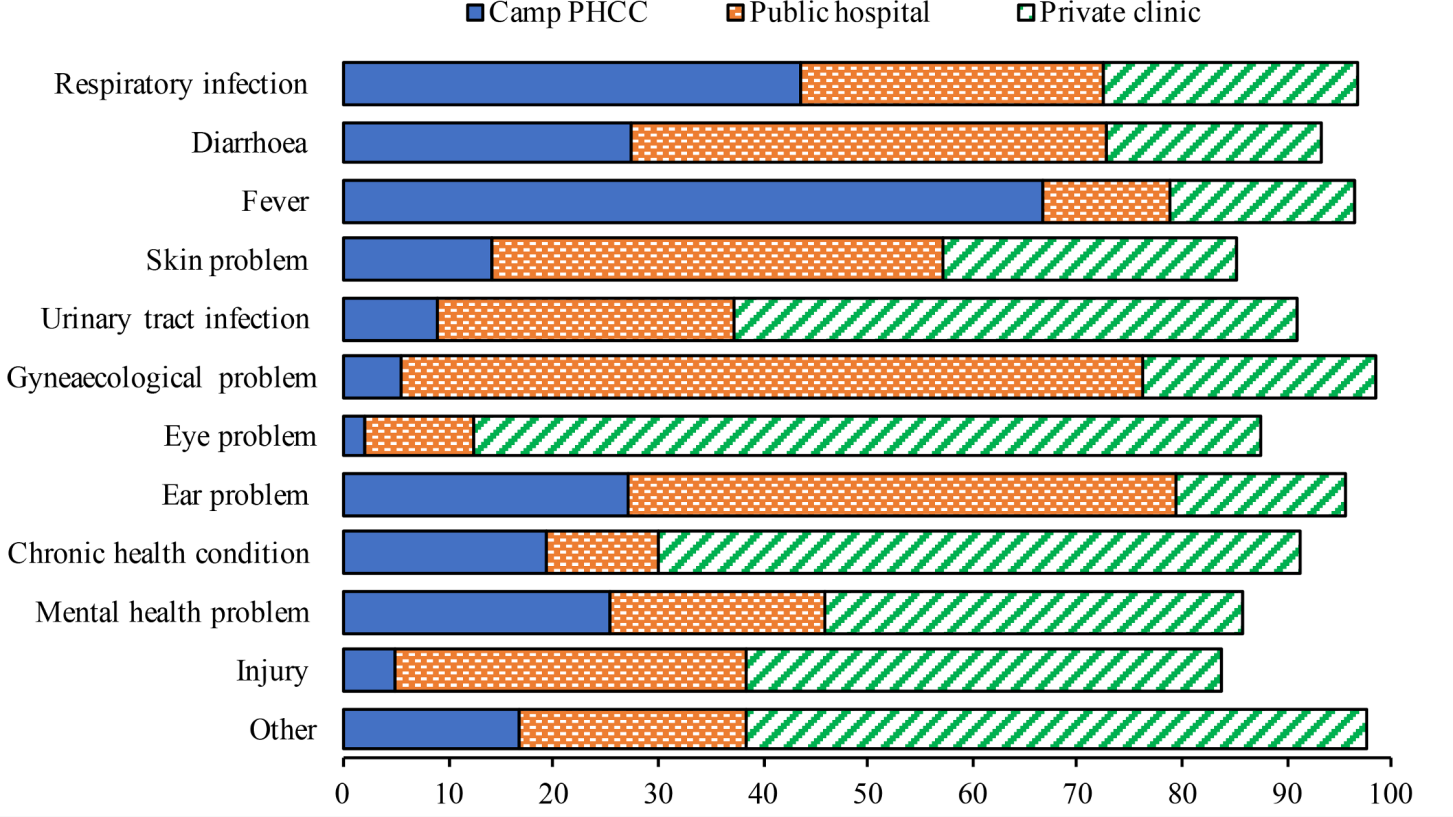
Care seeking in the two weeks preceding the survey.

**Fig 2:**
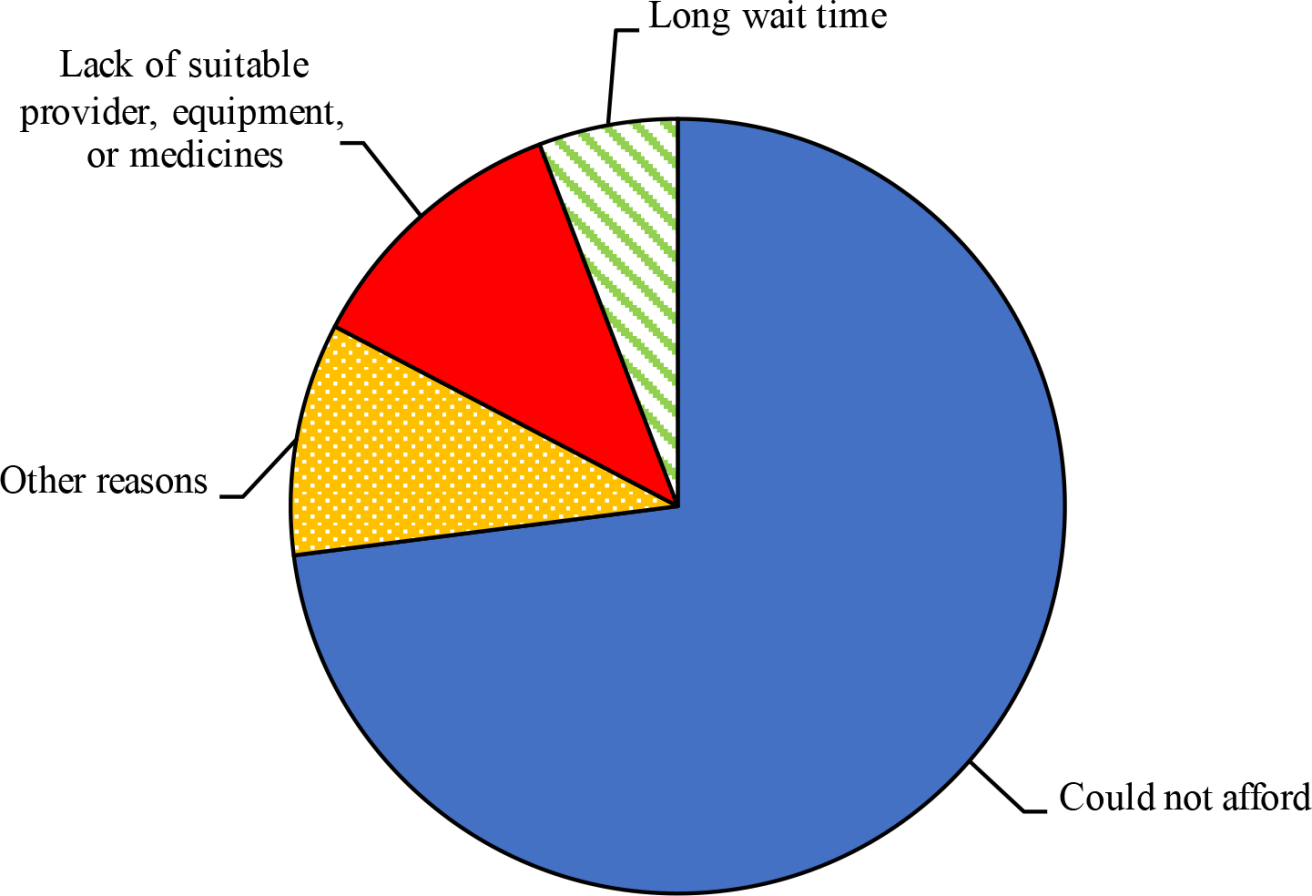
Reasons for not seeking care.

While households reported that all sick infants received care in a health facility, 8.4% (CI: 2.8–22.3) of young children, 8.4% (CI: 3.7–18.0) of older children, 9.0% (CI: 4.5–17.1) of male adults, 7.8% (CI: 4.6–12.9) of female adults and 4.1% (CI: 0.9–16.5) of older persons did not receive care outside the household for their health conditions (Table 2). Absence of care from the formal sector was particularly common among those suffering from an injury (16.3%; CI: 5.4–40.1), those with a skin problem (14.8%; CI: 4.2–40.6), and those with a mental health problem (14.3%; CI: 6.3–28.9) (Fig 1). Unaffordability was the main reason given for not seeking care (72.9%; CI: 55.3–85.5), followed by lack of suitable provider, equipment or medicines (11.5%; CI: 4.5–26.2) and long waiting time (5.8%; CI: 1.1–25.1) (Fig 2).

Most people sought care outside camps, either from a private clinic (41.8%; CI: 36.4–47.4) or a public hospital (27.3%; CI: 22.6–32.7). Only for fever (66.6%; CI: 44.8–83.1) and respiratory infections (43.5%; CI: 32.5–55.1) was the camp PHCC the most frequent choice among care seekers. Public hospitals were preferred in case of gynaecological problems (70.7%: CI: 41.5–89.1), ear problems (52.3%; CI: 22.6–80.5), diarrhoea (45.4%; CI: 31.1–60.4), and skin problems (43.1%; CI: 23.5–65.1). In all other cases, including eye problems (75.1%; CI: 51.5–89.6), chronic health conditions (61.4%; CI: 38.6–80.1), urinary tract infections (53.9%; CI: 39.2–68.0), injuries (45.3%; CI: 21.4–71.6) and mental health problems (39.7%; CI: 25.0–56.5), care was most commonly sought in private clinics (Fig 1). The mean out-of-pocket expenses varied significantly by type of health facility where care was sought (*p*<0.001). Among those who visited the camp PHCCs, the mean cost was 1.6 USD (CI: 0.6–2.5). In public hospitals the mean cost was 4.4 USD (CI: 2.3–6.6), while in private clinics it was 17.3 USD (CI: 14.3–20.2).

## Discussion

The increasingly complex burden of diseases among displaced populations, including communicable and non-communicable conditions as well as mental health problems and physical injuries, requires new health system strategies [3]. The household survey reported here provided a population-based assessment of the health needs and care seeking behaviours for a representative sample of Yazidis and other minority groups displaced by ISIS and currently living in camps in the Kurdistan Region of Iraq. The aim of the survey was to collect data that can help redesign health services for this population in an environment of growing demands and dwindling resources. Many of the issues identified, including burden of diseases and barriers to care, are common to the millions of displaced persons in the region.

In approximately one-third of interviewed households, one or more household members had needed care for some health conditions in the two weeks preceding the survey, the most likely to need care being older persons and infants. The reasons for needing care among infants and young children were predominately communicable diseases associated with poor living conditions. These findings are aligned with health assessments of Syrian refugee children in neighbouring countries as well as Iraqi IDP children in other parts of Iraq [13–15]. While communicable diseases remain common among adult and older persons, the prevalence of non-communicable diseases increases significantly with age. The high burden of non-communicable diseases among adult and older persons is also well documented in studies of Syrian refugees and is consistent with the ongoing regional demographic and life style changes [16–18]. Mental health problems were frequently reported as reason for needing care, particularly among adult women. This reflects the traumatic displacement experienced by Yazidis and other minorities and is aligned with previous findings of a high prevalence of post-traumatic stress disorder and major depression diagnosis among displaced Yazidis who crossed into Turkey [19]. Also consistent with previous studies, physical injuries were a commonly reported reason for needing care among adult men [16,17,20].

Over 90% of those with a health problem had sought care in the two weeks preceding the survey. This contrasts with the situation in central Iraq, where IDPs in informal settlements were reported to experience serious difficulties in accessing care [15]. The number of health facilities per population is higher in the Kurdistan Region than in other parts of Iraq [21], and the conflict-induced insecurity in Baghdad and Mosul has caused a migration of health workers to Kurdistan [22]. Better security in Kurdistan has also resulted in greater assistance from humanitarian agencies than in the rest of the country [23]. Data show that in the majority of cases care was sought in private clinics or directly in public hospitals rather than in the camp PHCCs. This reflects a common care seeking behaviour in Iraq, as the health system has historically been specialist-oriented and only recently have there been discussions of a stronger primary health care model [24]. As elsewhere in Iraq, private clinics are staffed by government doctors out-of-hours. More information is needed on why many displaced persons turn to the private sector, even when this implies a significant financial burden that can push households deeper into poverty, and why camp services remain underutilised.

Despite high levels of care seeking, cost was an important barrier to health service access. The reported mean out-of-pocket payment for care received was nearly 3 times higher in public hospitals than in the camp PHCCs and nearly 11 times higher in private clinics. Costs associated with the PHCCs were largely for medicines and tests not available in the PHCC. In addition, those seeking care outside camps had to face transportation costs. Although they were not recorded in the survey, transportation costs are not negligible as camps are mostly located in remote rural areas far from Duhok city and other main towns where public hospitals and private clinics are available. Cost-related barriers to care are likely to worsen as the ongoing financial crisis in the region has led the Kurdistan regional government to limit funding for the public health sector [25]. Delays and cuts in government employees’ salaries may weaken motivation among health providers. Doctors are likely to reduce the proportion of their time spent in public sector health facilities to further supplement their income through increased hours in private clinics.

With no end to this humanitarian crisis in sight, additional international support and an emphasis towards a stronger primary health care model are critical to ensure that the health needs of displaced persons are adequately addressed while limiting service costs. Building competencies of primary care doctors to care for common uncomplicated non-communicable diseases and other traditional specialist services, ensuring that essential medicines are adequately stocked in PHCC pharmacies, and establishing a referral system for medical complications are steps that can be taken. Creating strong health promotion programmes and utilising more health auxiliary staff at the primary care and community level can also be beneficial and reduce costs. Given the magnitude of the problem, community-based psychosocial rehabilitation should also be integrated in the primary health care services. With appropriate training, non-mental health personnel can be efficient in responding to the psychosocial distress of most displaced persons and detect those with severe trauma and chronic mental health disorders to be referred to specialist care. Finally, more information at the primary care level about the nature of injuries among adult men could guide development of injury prevention and management programmes.

## Conclusion

Communicable and non-communicable diseases as well as mental health problems and physical injuries were commonly reported reasons for needing care among displaced Yazidis and other minorities residing in BRHA camps. Although access to health services was good at the time of the survey, most households sought care from private clinics or public hospitals rather than from the camp PHCCs. Health status and access to care risk to deteriorate in the longer term, as funding to the public health system declines and the economic situation of in-camp households becomes even more tenuous. New strategies are required to address the health needs of displaced populations while limiting service costs.

## Abbreviations

BRHA: Board of Relief and Humanitarian Affairs
IDP: internally displaced person
ISIS: Islamic State of Iraq and Syria
